# Tomato–*Pseudomonas syringae* interactions under elevated CO_2_ concentration: the role of stomata

**DOI:** 10.1093/jxb/eru420

**Published:** 2014-10-21

**Authors:** Xin Li, Zenghui Sun, Shujun Shao, Shuai Zhang, Golam Jalal Ahammed, Guanqun Zhang, Yuping Jiang, Jie Zhou, Xiaojian Xia, Yanhong Zhou, Jingquan Yu, Kai Shi

**Affiliations:** ^1^Department of Horticulture, Zijingang Campus, Zhejiang University, 866 Yuhangtang Road, Hangzhou, 310058, China; ^2^Tea Research Insititute, Chinese Academy of Agricultural Science, Hangzhou, 310008, China; ^3^Zhuanghang Experimental Station, Shanghai Academy of Agricultural Sciences, 1000 Jinqi Road, Shanghai, 201403, China; ^4^Key Laboratory of Horticultural Plants Growth, Development and Quality Improvement, Agricultural Ministry of China, 866 Yuhangtang Road, Hangzhou, 310058, China

**Keywords:** Elevated CO_2_, nitric oxide, *Pseudomonas syringae*, *Solanum lycopersicum*, stomata.

## Abstract

Increasing atmospheric CO_2_ concentrations ([CO_2_]) in agricultural and natural ecosystems is known to reduce plant stomatal opening, but it is unclear whether these CO_2_-induced stomatal alterations are associated with foliar pathogen infections. In this study, tomato plants were grown under ambient and elevated [CO_2_] and inoculated with *Pseudomonas syringae* pv. *tomato* strain DC3000, a strain that is virulent on tomato plants. We found that elevated [CO_2_] enhanced tomato defence against *P. syringae*. Scanning electron microscopy analysis revealed that stomatal aperture of elevated [CO_2_] plants was considerably smaller than their ambient counterparts, which affected the behaviour of *P. syringae* bacteria on the upper surface of epidermal peels. Pharmacological experiments revealed that nitric oxide (NO) played a role in elevated [CO_2_]-induced stomatal closure. Silencing key genes involved in NO generation and stomatal closing, *nitrate reductase* (*NR*) and *guard cell slow-type anion channel 1* (*SLAC1*), blocked elevated [CO_2_]-induced stomatal closure and resulted in significant increases in *P. syringae* infection. However, the *SLAC1*-silenced plants, but not the *NR*-silenced plants, still had significantly higher defence under elevated [CO_2_] compared with plants treated with ambient [CO_2_]. Similar results were obtained when the stomata-limiting factor for *P. syringae* entry was excluded by syringe infiltration inoculation. These results indicate that elevated [CO_2_] induces defence against *P. syringae* in tomato plants, not only by reducing the stomata-mediated entry of *P. syringae* but also by invoking a stomata-independent pathway to counteract *P. syringae*. This information is valuable for designing proper strategies against bacterial pathogens under changing agricultural and natural ecosystems.

## Summary

Elevated CO_2_ enhanced defence against *Pseudomonas syringae* in tomato plants, not only by reducing stomata-mediated entry of *P. syringae* but also by invoking a stomata-independent pathway to counteract *P. syringae*.

## Introduction

In modern greenhouse cultivation, CO_2_ enrichment is considered as an important means of increasing the yield and quality of agricultural produce, particularly in C_3_ horticultural crops (Uprety, 1998; Dion *et al.*, 2013). A steady increase in atmospheric CO_2_ levels has been observed over the past 150 years, and this increase is projected to continue so that CO_2_ levels will be doubled over the next 50 years (Levine *et al.*, 2008). The rising CO_2_ concentration ([CO_2_]) has influenced recent research interests, and a strong emphasis has been placed on the impact of elevated [CO_2_] on photosynthesis and plant growth in the era of climate change (Long *et al.*, 2004; Levine *et al.*, 2008). Despite this trend, the effects of rising atmospheric [CO_2_] on plant–pathogen interactions have received little attention. Studies conducted in free-air CO_2_ enrichment sites, open-top chambers, and growth chambers involving plant diseases have shown that responses to elevated [CO_2_] may vary with the host–pathogen system, and thus the severity and/or incidence of disease may either increase, decrease, or remain unaffected (Eastburn *et al.*, 2010; Melloy *et al.*, 2010; West *et al.*, 2012). As plant–pathogen interactions under increasing [CO_2_] have the potential to interrupt both agricultural and natural systems significantly, additional work is required to understand the extent and mechanisms through which elevated [CO_2_] affects plant diseases. Such studies will be essential for making accurate predictions regarding future plant disease dynamics and proper management of agricultural and natural ecosystems under changing climatic conditions.

Plants have a complex array of defence mechanisms. For example, the cell wall is covered with a waxy cuticle that serves as a potent physical barrier. Although some pathogenic fungi infect plants by penetrating the cell wall, many foliar bacterial plant pathogens invade plants primarily through natural surface openings, namely, through the stomata (Kumar *et al.*, 2012). Stomata are the small pores in the leaf epidermis, formed by a pair of guard cells that have developed mechanisms to sense and respond to various endogenous and environmental stimuli. By changing the size of the stomatal pore, stomata can regulate gas exchange between the plant and environment, as well as control water loss (Melotto *et al.*, 2008). Manning and Vontiedemann (1995) speculated that reducing the stomatal conductance and the size of the stomatal aperture could inhibit the entry of bacterial pathogens through the stomata. Notably, reduced stomatal conductance is one of the primary effects of rising atmospheric [CO_2_] on plants (Drake *et al.*, 1997; Long *et al.*, 2004); however, it is not clear whether the CO_2_-induced alterations in the stomatal characteristics are associated with foliar bacterial pathogenic infections. In a recent study involving oilseed *Brassica juncea*, the decrease in the disease index of downy mildew caused by the stomata-invading pathogen *Hyaloperonospora brassicae* was suggested to be associated with a decrease in stomatal density, pore size, and stomatal conductance in response to elevated [CO_2_] (Mathur *et al.*, 2013). Similarly, in *Populus*, the clones that opened their stomata late in the morning were more resistant to *Melampsora larici-populina* Kleb., compared with those that opened their stomata earlier (Siwecki and Przybyl, 1981). By contrast, there are also reports suggesting that stomatal conductance and stomatal density are not correlated with pathogenic infection (Riikonen *et al.*, 2008; O’Keefe *et al.*, 2013). Furthermore, elevated [CO_2_] not only reduces stomatal conductance but also stimulates higher rates of photosynthesis (Drake *et al.*, 1997; Ainsworth and Rogers, 2007). The altered leaf chemistry, including elevated levels of carbon gain or secondary compounds, also has the potential to affect disease severity (McElrone *et al.*, 2005; Ghasemzadeh and Jaafar, 2011; Huang *et al.*, 2012). The dependence of foliar bacterial disease on stomatal conductance or the leaf chemistry under elevated [CO_2_] has yet to be elucidated.

*Pseudomonas syringae* pv. *tomato* DC3000 (*P. syringae*) is a Gram-negative bacterium capable of infecting tomato and *Arabidopsis*. This bacterium is a model pathogen used to investigate the molecular mechanisms underlying plant–pathogen interactions (Whalen *et al.*, 1991; Preston, 2000; Zeng *et al.*, 2011). Furthermore, *P. syringae* is also considered as one of the stomata-invading pathogens (Melotto *et al.*, 2008). Elucidating how elevated [CO_2_] affects host symptoms will advance our current understanding of the dynamics between plants and pathogens in future natural ecosystems. Therefore, the present study was conducted with the following objectives: (i) to examine whether elevated [CO_2_] has positive effects on plant defence against *P. syringae* infection, and if so, (ii) to identify the mechanism involved. In the present study, tomato seedlings were grown under ambient and elevated [CO_2_] and inoculated with *P. syringae* pv. *tomato* DC3000. Using pharmacological and gene silencing approaches, we obtained evidence that elevated [CO_2_] induces resistance to *P. syringae* in tomato plants. In addition to reducing the stomata-mediated pathogen entry of *P. syringae*, elevated [CO_2_] also invokes stomata-independent pathways to counteract *P. syringae*.

## Materials and methods

### Plants, CO_2_, bacteria, and chemical treatment

Tomato (*Solanum lycopersicum* L. cv. *Zheza 205*) seeds were sown in plastic pots (15cm diameter) filled with a 7:3 (v/v) mixture of peat and vermiculite. The pots were placed under the following conditions: a 14-h photoperiod, day/night temperature of 25/20 °C, and light intensity of 600 µmol m^–2^ s^–1^. The plants were watered daily with Hoagland’s nutrient solution. The six-stage seedlings were transferred to controlled environment cabinets (Conviron, Winnipeg, Canada), where the atmospheric [CO_2_] was maintained at either 380 µmol mol^–1^ or 800 µmol mol^–1^, corresponding to the “ambient [CO_2_]” and “elevated [CO_2_]”, respectively. The elevated CO_2_ concentration of 800 µmol mol^–1^ has also been widely used in previous studies (Melloy *et al.*, 2010; Huang *et al.*, 2012). After 48h, plants exposed to both ambient and elevated [CO_2_] were subjected to *P. syringae* inoculation or chemical spray treatment of 200 µM sodium nitroprusside [SNP, nitric oxide (NO) donor], 500 µM 2-(4-carboxyphenyl)-4,4,5,5-tetramethylimidazoline-1-oxyl-3-oxide potassium salt (cPTIO, NO scavenger), or 0.05 µM coronatine (COR); distilled water served as control. In the experiments employing a combined treatment of *P. syringae* and chemicals, the chemical pre-treatments were applied to the tomato leaves 6h prior to *P. syringae* inoculation.

### Pathogen inoculation and bacterial growth analysis

The bacteria *P. syringae* pv. *tomato* DC3000 was cultured in King’s B medium containing rifampicin (25mg ml^–1^) at 28 °C. A single bacterial colony was cultured at 28 °C with shaking until log phase. Then, the bacterial cells were collected by centrifugation at 4000 *g* for 10min and resuspended in 10mM MgCl_2_ until an OD_600_ of 0.2 was reached, which corresponded to approximately 10^8^ colony-forming units (cfu) ml^−1^. The inoculation was carried out by spraying the bacterial suspension on the whole plant at a final concentration of 10^6^ cfu ml^–1^, which was obtained by serial dilution, according to Katagiri *et al.* (2002). The MgCl_2_ buffer was applied as a mock inoculation. The bacterial populations were measured from four leaves per plant at 48 hours post-inoculation (hpi), according to the method described previously (Wolfe *et al.*, 2000).

To exclude the factor for *P. syringae* entry through stomata, *P. syringae* was artificially infiltrated directly into the leaf apoplast using 1ml needleless syringe at a final concentration of 10^5^ cfu ml^– 1^. At 4 days after inoculation, disease symptoms were assessed using a chlorophyll fluorescence imaging system (Liao *et al.*, 2013). Generally, in actinic light (300µmol m^−2^ s^−1^), maximal fluorescence (*F*_m_′) and steady-state fluorescence prior to the flash (*F*) were measured while saturated light flashes were applied every 20s, and the photochemical quantum yield at photosystem II (ΦPSII) of light-adapted leaves was calculated as *F*_m_′–*F*/*F*_m_′.

### Measurement of stomatal conductance and aperture

Stomatal conductance was measured using an infrared gas analyser-based portable photosynthesis system (LI-6400; LI-COR, Lincoln, NE, USA). The air temperature, relative humidity, and light intensity were maintained at 25 °C, 85%, and 1000 µmol m^–2^ s^–1^, respectively. The CO_2_ concentration was set to match the growth CO_2_ concentration, i.e., 380 and 800 µmol mol^–1^ for ambient and elevated [CO_2_]-treated plants, respectively.

To view the stomatal aperture by light microscopy, abaxial epidermises were peeled with forceps and floated on buffer (10mM MES, 30mM KCl, 0.5mM Ca^2+^, pH 6.2). The temperature during the experiment was set at 25 °C, and the stomatal apertures were measured using a light microscopy equipped with a digital camera (Leica Microsystems, Wetzlar, Germany), as well as the image analysis software Adobe Photoshop CS5 (Adobe, San Jose, CA, USA).

Samples for scanning electron microscopy (SEM) were prepared following standard procedures (Singh *et al.*, 2000). Leaf samples were immersed in 5% glutaraldehyde overnight, washed in 0.1M phosphate buffer (pH 7.0), then treated in 1% osmium tetroxide for 2h. The samples were dehydrated using serially diluted ethanol (70% to 100%) and iso-amyl acetate. Specimens were dried in a Hitachi HCP-2 critical point dryer (Tokyo, Japan) with liquid CO_2_ and mounted onto double-coated carbon conductive tapes that were attached onto specimen holders. Samples were sputter-coated with gold-platinum and examined in a Hitachi TM-1000 TSEM (Tokyo, Japan).

### Quantification of NO content and trypan blue staining

The NO accumulation was assayed and calculated by following the conversion of oxyhaemoglobin (HbO_2_) to methaemoglobin (MetHb) spectrophotometrically at 401nm and 421nm as described by Pasqualini *et al.* (2009). Briefly, samples corresponding to 0.3g of fresh leaf tissue were ground in a mortar and homogenized in 100mM phosphate buffer (pH 7.0) and 0.6% (w:v) insoluble polyvinylpolypyrrolidone. The extracts were clarified by adding powdered activated carbon and centrifuged at 10 000 *g* for 10min at 4 °C. The supernatant was filtered through a PTFE Millipore membrane (0.45 µm) and immediately assayed for NO. Five minutes before HbO_2_ addition, samples were pre-treated with catalase (100U) and superoxide dismutase (100U) to remove reactive oxygen species.

Trypan blue staining was performed as described by Bai *et al.* (2012). Tomato leaves were boiled for 10min in the lactophenol-trypan blue solution (10ml of lactic acid, 10ml of glycerol, 9.3g of phenol, 10mg of trypan blue, dissolved in 10ml of distilled water) and then decolourized in chloral hydrate (25g chloral hydrate dissolved in 10ml distilled water) for 12h. The dark-brown spots were photographed to evaluate cell death.

### Virus-induced gene silencing

The tobacco rattle virus (TRV) virus-induced gene silencing (VIGS) constructs used for the silencing of the tomato *nitrate reductase* (*NR*) and *guard cell slow-type anion channel 1* (*SLAC1*) gene were generated by cloning a 379-bp *NR* cDNA fragment and 430-bp *SLAC1* cDNA fragment, which were PCR-amplified using the following primers: forward primer 5ʹ CGgaattcAGACCCTCAACACTCAACCC 3ʹ and reverse primer 5ʹ CGggatccCAGAATGCTGTCAACACCAC 3ʹ (with *Eco*RI and *Bam*HI restriction sites) for *NR*; forward primer 5ʹ CGCggatccTGATGAATTTGATGGCTTGG 3ʹ and reverse primer 5ʹ TGCtctagaGGCGCACTCTTTGTCTCC 3ʹ (with *Bam*HI and *Xba*I restriction sites) for *SLAC1*. The amplified fragments were digested with restriction enzymes and ligated into the corresponding restriction sites of pTRV2. An empty pTRV2 vector was used as a control. The resulting plasmids were transformed into *Agrobacterium tumefaciens* strain GV3101 and were grown for 16–18h in LB broth supplemented with the appropriate antibiotics (Liu *et al.*, 2002). The cells were pelleted, washed, and resuspended in infiltration buffer [10mM MgCl_2_, 10mM 2-(*N*-morpholine)-ethane-sulphonic acid (MES), pH 5.5, 150mM acetosyringone). Three hours after induction at room temperature, *A. tumefaciens* containing pTRV1 and pTRV2 (empty vector or with target gene insert) were mixed in a 1:1 ratio to a final OD_600_ of 0.6. Leaves and cotyledons of 2-week-old tomato seedlings were infiltrated using a blunt syringe. Plants were then kept at 21 °C for 4 weeks before they were used for the experiments. The silencing efficiency was assessed by quantitative real-time PCR as described in previous studies (Livak and Schmittgen, 2001).

### Statistical analysis

At least four independent replicates were conducted for each determination. The data were subjected to analysis of variance using SAS 8.0 software package (SAS Institute, Cary, NC), and the means were compared using Tukey’s test at the *P*<0.05 level.

## Results

### Response of stomatal movement to *P. syringae* in ambient and elevated [CO_2_]

**Fig. 1.**
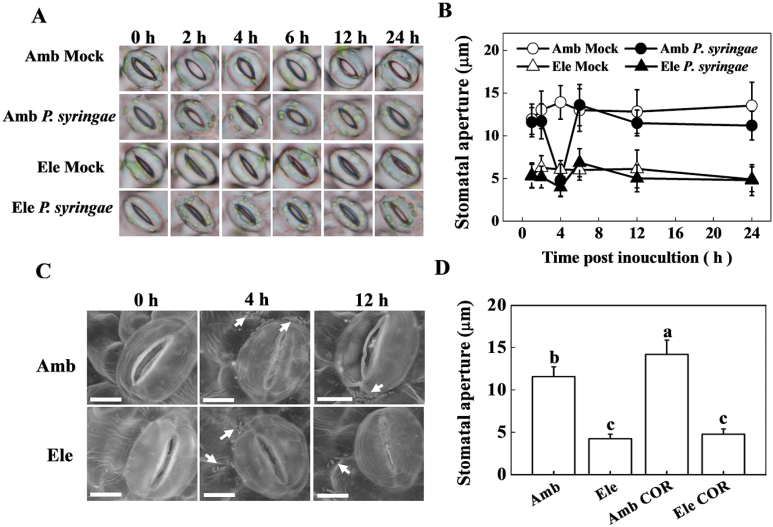
Effects of *Pseudomonas syringae* and coronatine (COR) spray treatment on the stomatal aperture of tomato leaves grown at either ambient (380 µmol mol^–1^) or elevated (800 µmol mol^–1^) CO_2_ concentration. (A) Representative light microscopy image of stomata in response to *P. syringae* infection at the indicated time post inoculation; the stomatal aperture data are shown in B. Values are the means±SD (*n=*25). (C) Representative scanning electron microscopy image of stomata in response to *P. syringae* infection at the indicated time post inoculation. A *P. syringae* inoculum is indicated as an arrow in each image. Scale bar=25 µm. (D) Stomatal aperture in response to exogenous COR application; leaf samples were collected 4h post-chemical application. Values are the means±SD (*n=*25). Different letters depict significant differences between the treatments (*P*<0.05). Tomato plants were grown in ambient or elevated CO_2_ conditions for 48h followed by *P. syringae* or COR spray treatment. (This figure is available in colour at *JXB* online.)

After 48h of elevated [CO_2_] pre-treatment (800 µmol mol^–1^), the average stomatal aperture of the tomato leaves was much smaller than that of their ambient counterparts (Fig. 1A, B). Upon *P. syringae* spray inoculation, the stomatal openings of ambient [CO_2_]-treated leaves decreased rapidly in epidermal peels. At 4 hpi, the average stomatal aperture of the leaves decreased to the lowest level, which was only 34.3% of the average stomatal aperture of the mock-inoculated plants grown under ambient [CO_2_]. The *P. syringae*-induced stomatal closure in ambient [CO_2_]-treated leaves was only transient because the stomatal aperture recovered to mock levels at 6 hpi and remained constant thereafter. By contrast, under elevated [CO_2_], *P. syringae* spray inoculation did not lead to evident changes in stomatal movement, and the stomatal aperture of the inoculated plants was much smaller than that of the ambient counterpart but approximately the same as that of the mock-inoculated and elevated [CO_2_]-treated plants (Fig. 1A, B).

The distribution of bacteria on the surface of tomato epidermal cells in response to [CO_2_] elevation and *P. syringae* spray inoculation was observed by SEM (Fig. 1C). Twenty five stomata from at least 4 plant leaves for each treatment were observed, and there were general observations that elevated [CO_2_]-treated plants had a smaller average stomatal aperture than that of their ambient [CO_2_] counterparts and showed a transient closing of their stomata at 4 hpi, which is in accordance with the results obtained by light microscopy. The bacterial inoculum flocked together near the guard cells at 4 hpi, irrespective of CO_2_ concentration treatment. Thereafter, following the reopening of the stomata, the bacteria remained distributed around the open stomata in ambient [CO_2_] but not in elevated [CO_2_]-treated plants. By contrast, the bacteria remained scattered on the surface of tomato epidermal cells of leaves under the elevated [CO_2_] treatment.

As *P. syringae* can produce polyketide toxin COR to reopen stomata, we examined whether elevated CO_2_ was preventing the reopening of stomata by disrupting the function of COR (Fig. 1D). Indeed, after 4h of COR treatment, COR slightly but significantly increased stomatal aperture sizes in ambient [CO_2_]-treated plants, whilst such effects were not observed in elevated [CO_2_]-treated plants.

### Involvement of NO in elevated [CO_2_]-induced stomatal movement

**Fig. 2.**
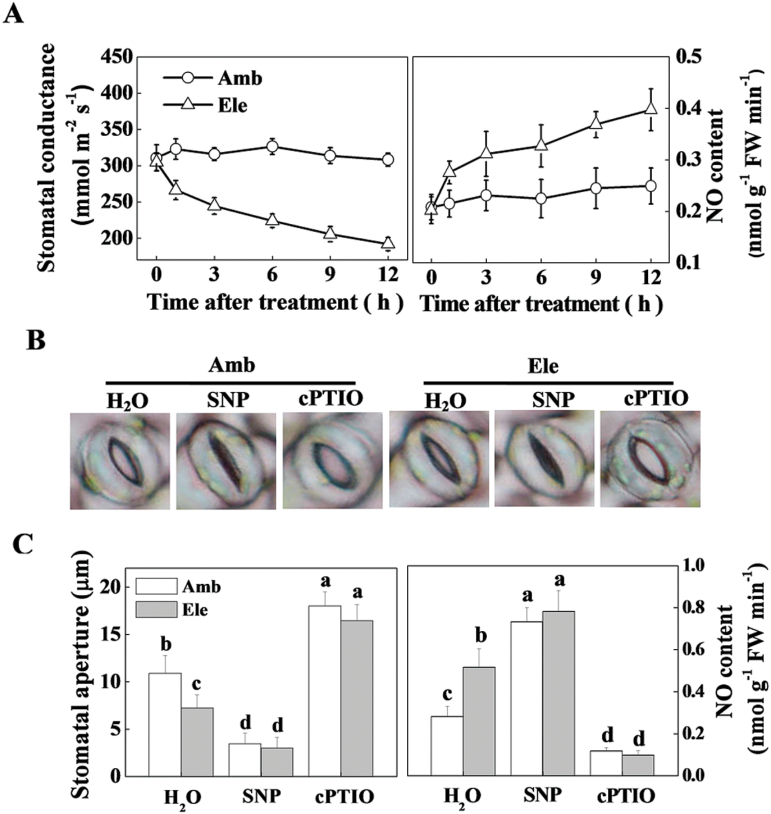
The involvement of nitric oxide (NO) in elevated CO_2_-induced stomatal closure. (A) Time course of the changes in stomatal conductance and NO content of tomato leaves grown at either ambient (380 µmol mol^–1^) or elevated (800 µmol mol^–1^) CO_2_ concentrations. Values are the means±SD (*n=*6). (B, C) Effects of exogenous NO modulators on the stomatal aperture and endogenous NO content of tomato leaves grown at either ambient or elevated CO_2_ concentration: (B) Representative image of stomata. (C) Stomatal aperture data (left panel) and NO content (right panel). Tomato plants were grown in ambient or elevated CO_2_ conditions for 48h followed by H_2_O, SNP, or cPTIO treatment, and leaf samples were collected 6h post-chemical application. Values are the means±SD, *n=*25 for the stomatal aperture data and *n*=4 for the NO content. Different letters depict significant differences between the treatments (*P*< 0.05). (This figure is available in colour at *JXB* online.)

Compared with ambient [CO_2_], plants treated with elevated [CO_2_] showed a rapid and sharp decrease in stomatal conductance, which was accompanied by a simultaneous increase in endogenous NO content (Fig. 2A). At 12h after treatment with elevated [CO_2_], the stomatal conductance had decreased by 37.7%, whereas the NO content increased by 59.2%. After 48h of ambient or elevated [CO_2_] treatment, exogenous pharmacological SNP (NO donor) and cPTIO (NO scavenger) were applied to the tomato leaves to study the role of NO in [CO_2_]-regulated stomatal movements (Fig. 2B, C). As expected, the endogenous NO content was either increased by exogenous SNP or decreased by cPTIO (Fig. 2C). SNP further decreased the stomatal aperture of plants grown in both ambient and elevated [CO_2_] conditions, whereas cPTIO application fully compromised the elevated [CO_2_]-induced stomatal closure, suggesting that NO plays a role in the elevated [CO_2_]-induced stomatal closure (Fig. 2B, C).

### Effect of exogenous NO modulator on *P. syringae* infection in ambient and elevated [CO_2_] conditions

**Fig. 3.**
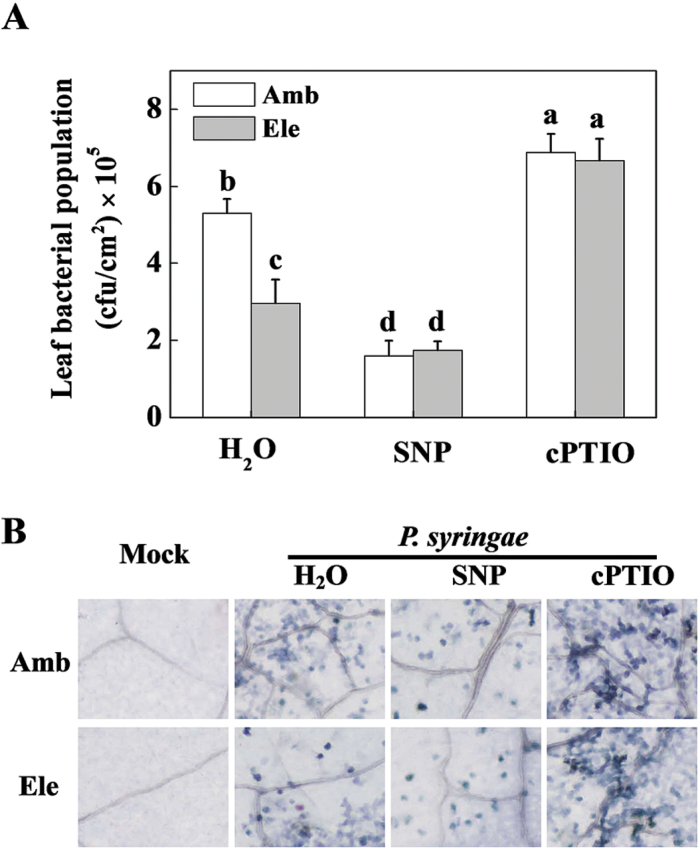
Effects of exogenous nitric oxide (NO) modulators on *Pseudomonas syringae* disease expression in tomato leaves grown at either an ambient (380 µmol mol^–1^) or an elevated (800 µmol mol^–1^) CO_2_ concentration. (A) Bacterial population of the tomato leaves; values are the means±SD (*n=*10). Different letters depict significant differences between the treatments (*P*< 0.05). (B) Trypan blue staining for cell death. Tomato plants were exposed to ambient or elevated CO_2_ for 48h, then treated with H_2_O, 200 µM SNP, or 500 µM cPTIO, and spray inoculated with *P. syringae* 6h later. Leaf samples were collected at 48h post-inoculation.

As NO plays a role in the elevated [CO_2_]-induced stomatal closure, we studied the effects of NO on plant innate immunity against *P. syringae* under conditions of both ambient and elevated [CO_2_] (Fig. 3). We pre-treated the tomato leaves of both ambient and elevated [CO_2_] plants with SNP and cPTIO before spray inoculating them with *P. syringae*. We found that elevated [CO_2_] effectively alleviated the bacterial infection, and the bacterial population growth on plants under elevated [CO_2_] was reduced to 56.0% compared with those plants grown in the ambient [CO_2_] at 48 hpi. The NO scavenger cPTIO fully blocked elevated [CO_2_]-induced alleviation in *P. syringae* infection, whereas SNP application on the plants significantly reduced the bacterial infection in both the ambient and elevated [CO_2_] plants. Under SNP-pre-treated condition, the bacterial population of *P. syringae* was reduced to a similar level in both the ambient and elevated [CO_2_] plants.

### Effects of *NR* and *SLAC1* gene silencing on NO content, stomatal aperture, and *P. syringae* infection

**Fig. 4.**
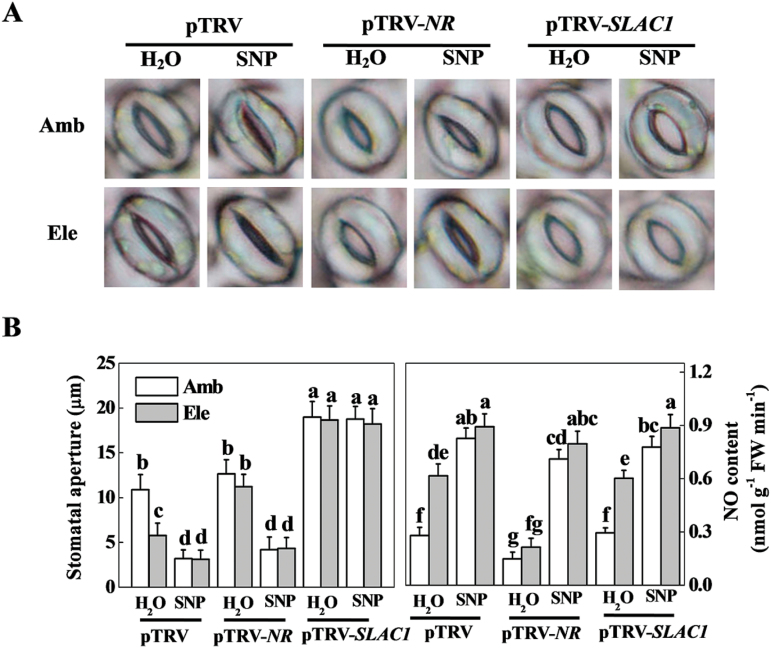
Effects of gene silencing (*NR* and *SLAC1)* and an exogenous nitric oxide (NO) donor on the stomatal aperture and endogenous NO content of tomato leaves grown at either ambient (380 µmol mol^–1^) or elevated (800 µmol mol^–1^) CO_2_ concentration. (A) Representative image of stomata. (B) Stomatal aperture data (left panel) and NO content (right panel). pTRV control-, *NR*- and *SLAC1*-silenced tomato plants were grown in ambient or elevated CO_2_ conditions for 48h followed by H_2_O or SNP treatment, and leaf samples were collected 6h post application. Values are the means±SD, *n*=25 for the stomatal aperture data and *n*=4 for the NO content. Different letters depict significant differences between the treatments (*P*<0.05). (This figure is available in colour at *JXB* online.)

To verify the role of elevated [CO_2_]-induced stomatal closure and NO generation in basal defence against *P. syringae* spray inoculation, the key genes involved in stomatal closure and NO generation, i.e. *SLAC1* and *NR*, respectively, were silenced via VIGS in tomato plants. Under ambient [CO_2_], neither stomatal aperture nor endogenous NO content was affected by *NR* silencing (Fig. 4). Under elevated [CO_2_], *NR* silencing significantly reduced elevated [CO_2_]-induced NO accumulation and subsequent stomatal closure; both were reversed by exogenous SNP application. By contrast, *SLAC1* gene silencing significantly increased the stomatal aperture in both ambient and elevated [CO_2_], although it had no evident effect on endogenous NO content. Exogenous SNP did not seem to affect the stomatal aperture in *SLAC1*-silenced plants, even though the endogenous NO concentration was significantly elevated in these plants (Fig. 4).

**Fig. 5.**
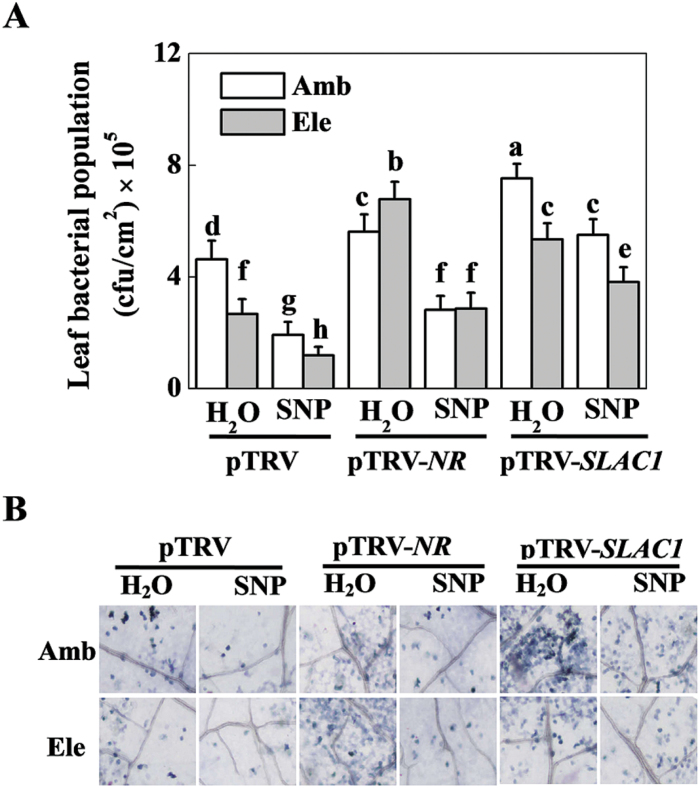
Effects of gene silencing (*NR* and *SLAC1)* and an exogenous nitric oxide (NO) donor on *Pseudomonas syringae* disease expression in tomato leaves grown at either ambient (380 µmol mol^–1^) or elevated (800 µmol mol^–1^) CO_2_ concentration. (A) Bacterial population of the tomato leaves. Values are the means±SD (*n=*10). Different letters depict significant differences between the treatments (*P*<0.05). (B) Trypan blue staining for cell death. pTRV control-, *NR*- and *SLAC1*-silenced tomato plants were exposed to ambient or elevated CO_2_ for 48h, then treated with H_2_O or 200 µM SNP, and spray inoculated with *P. syringae* 6h later. Leaf samples were collected at 48h post-inoculation.

The elevated [CO_2_]-induced *P. syringae* defence response also changed in conjunction with the *SLAC1*- and *NR*-silencing effects on the stomatal aperture and NO content (Fig. 5). In ambient [CO_2_], we observed that both the *NR*- and *SLAC1-*silenced plants, especially the pTRV-*SLAC1* plants, were more sensitive to the infection of bacteria and exhibited significantly higher bacterial colony counts and cell death at 48 hpi. The elevated [CO_2_]-induced *P. syringae* defence response was blocked by *NR-*silencing. However, in *SLAC1*-silenced but not *NR-*silenced plants, elevated [CO_2_] still had significantly lower bacterial growth population compared with the ambient [CO_2_] plants. Under both ambient and elevated [CO_2_], exogenous SNP significantly reduced the bacterial population of all three plant materials, although the alleviation effect was smaller in pTRV-*SLAC1* compared with pTRV and pTRV-*NR* plants.

### Effect of elevated [CO_2_] on *P. syringae* infection under syringe-inoculation condition

**Fig. 6.**
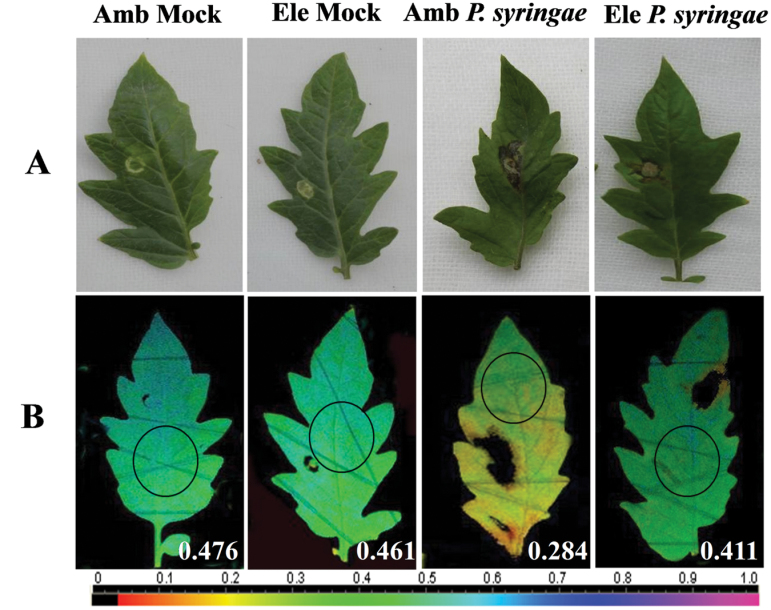
Effects of *Pseudomonas syringae* syringe infiltration inoculation on tomato leaves grown at either ambient (380 µmol mol^–1^) or elevated (800 µmol mol^–1^) CO_2_ concentration. (A) Photographed leaf disease symptoms. (B) Representative image of leaf photochemical quantum yield at photosystem II (ΦPSII). The circles in the images indicate the locations where the fluorescence measurements data were collected, and these data are shown in each figure. The colour gradient scale below B indicates the magnitude of the fluorescence signal represented by each colour. Tomato plants were grown in ambient or elevated CO_2_ conditions for 48h followed by *P. syringae* syringe infiltration inoculation, and data were collected at 4 days post inoculation.

To exclude the stomata-limiting factor for entry of bacterial inoculum, *P. syringae* was also inoculated into leaf interior by needleless syringe infiltration method after 48h of ambient or elevated [CO_2_] pre-treatment. As pathogen infection often results in a reduction in the operating efficiency of PSII, the chlorophyll fluorescence imaging method was used to analyse the response of ΦPSII to *P. syringae* infection (Fig. 6). In absence of restriction in stomatal movement, elevated [CO_2_]-treated leaves still exhibited remarkably higher defence against *P. syringae* than ambient [CO_2_]-treated leaves which was evident by minor spread of *P. syringae* lesions in elevated [CO_2_]-treated leaves at 4 days post inoculation (Fig. 6A). Furthermore, elevated [CO_2_]-treated plants also had higher ΦPSII than that of ambient [CO_2_] counterpart (Fig. 6B).

## Discussion

Increasing atmospheric [CO_2_] is creating novel environment for plants and is likely to have significant consequence with regard to the relationship between plant pathogens and their hosts. Previous studies on the effects of elevated [CO_2_] on plant–pathogen interactions have produced conflicting results (Lake and Wade, 2009; Newton *et al.*, 2011). For example, the plant fungal pathogen *Colletotrichum gloeosporiodes* exhibited increased fecundity and aggressiveness over 25 infection cycles in the host *Stylosanthes scabra* under elevated [CO_2_] (Chakraborty and Datta, 2003). However, investigations into the systemic responses of tomato to *Tomato yellow leaf curl virus* (TYLCV) and of tobacco to *Potato virus Y* found that elevated [CO_2_] decreased disease incidence and severity (Matros *et al.*, 2006; Huang *et al.*, 2012). As a model pathogen, *P. syringae* has been well studied; however, the impacts of elevated [CO_2_] on *P. syringae* diseases are largely unknown. The present study is among the first to examine the effects of elevated [CO_2_] on host symptoms associated with *P. syringae* infection. Generally, elevated [CO_2_] enhanced plant resistance to infection with *P. syringae* via a dual mechanism. This information is helpful for predicting the responses of bacterial pathogens to elevated atmospheric [CO_2_].

Pathogen entry into hosts is a critical step before the onset of the infection and disease progression. There is evidence indicating that *P. syringae* bacteria use the leaf surface stomata for entry into the host tissue (Boureau *et al.*, 2002). Reduced stomatal opening in response to elevated atmospheric [CO_2_] has previously been well documented (Drake *et al.*, 1997). In this study, we investigated whether stomatal closure mediated the defence effects against *P. syringae* in response to elevated [CO_2_]. Based on the light microscopy and SEM observations, we noticed that the ambient [CO_2_]-treated leaves exhibited a transient stomatal closing in response to *P. syringae* spray inoculation at approximately 4 hpi (Fig. 1). Similarly, *A. thaliana* stomata close in response to live bacteria and purified pathogen/microbe-associated molecular patterns (PAMPs/MAMPs) in a solution system; thus, Melotto *et al.* (2006) suggested that stomata played an active role in restricting bacterial invasion as part of the innate immune response, but not only as a passive port of entry. It has also been reported that bacterium-induced stomatal closure requires PAMPs signalling and homeostasis of the defence hormone salicylic acid, and is upstream of signalling regulated by abscisic acid in the guard cell (Melotto *et al.*, 2006; Melotto *et al.*, 2008). In addition, it seems that NO is also required for PAMPs and bacteria to close stomata, as NO production in guard cells could also be rapidly induced by the known MAMPs, flagellin (flg22), or lipopolysaccharide (LPS), and inhibitor of NO synthase could effectively block flg22-, LPS-, and *E. coli*-induced stomatal closure (Melotto *et al.*, 2006). To counter host defences during infection and in the apoplast, *P. syringae* and other plant pathogenic bacteria have evolved a variety of virulence factors to subvert host defences or to obtain nutrients (Nomura and He, 2005). *P. syringae* pv. *tomato* DC3000, used in this study, can produce polyketide toxin COR to promote stomatal opening and disrupts plant defence responses (Melotto *et al.*, 2006; Zeng *et al.*, 2011). The stomatal movement in the *P. syringae*-inoculated ambient [CO_2_] plants in this study (Fig. 1) was in accordance with the model proposed in previous studies (Melotto *et al.*, 2006; Melotto *et al.*, 2008). By contrast, under elevated [CO_2_], the stomatal aperture was constantly smaller than the ambient counterpart and did not show any evident transient changes in response to *P. syringae* inoculation (Fig. 1A–C). Furthermore, COR-induced stomatal opening was also effectively counteracted by elevated [CO_2_] compared with an ambient counterpart (Fig. 1D), which might partly contribute to tomato defence against *P. syringae* infection under elevated [CO_2_]. These differences in *P. syringae*- and COR-induced stomatal movement between ambient and elevated [CO_2_] treatment may contribute to the different behaviour of *P. syringae* bacteria in these plants; namely, the concentrated distribution of *P. syringae* around the reopened stomata in ambient [CO_2_]-treated plants might allow pathogens to enter the intercellular space more successfully compared with the elevated [CO_2_] plants, in which bacteria were dispersed on the surface of tomato epidermal cells among the closed stomata (Fig. 1).

In the present study, the elevated [CO_2_]-induced rapid decrease in stomatal conductance was accompanied by a simultaneous increase of endogenous NO content (Fig. 2). Furthermore, exogenous application of the NO donor SNP further decreased stomatal aperture, whereas its scavenger cPTIO fully abolished elevated [CO_2_]-induced stomatal closure (Fig. 2). It seems that NO plays a pivotal role in elevated [CO_2_]-induced guard cell movement and stomatal closure. In previous studies, an NO-induced Ca^2+^ increase and subsequent stomatal closure have been observed in the guard cells of *Arabidopsis* and *Vicia faba* (Neill *et al.*, 2002; Garcia-Mata and Lamattina, 2003). NO has also been demonstrated to be a signalling intermediate during bicarbonate-induced (mimic of elevated [CO_2_]) stomatal closure in *Pisum sativum* (Kolla and Raghavendra, 2007). The elevated [CO_2_]-induced and NO-mediated stomatal closure might in turn participate in the defence against *P. syringae* infection. To verify and extend this knowledge *in planta*, pharmacological and gene silencing approaches were used to control stomatal opening, and the mechanisms of elevated [CO_2_]-induced defence against *P. syringae* were investigated accordingly. In correspondence with the involvement of NO in stomatal closing, elevated [CO_2_]-induced *P. syringae* defence was fully abolished by cPTIO but enhanced by SNP (Fig. 3). Previous studies have demonstrated that NR is the key enzyme in the reduction of nitrite to NO in several physiological contexts, including stomatal closure (Rockel *et al.*, 2002; Bright *et al.*, 2006; Bellin *et al.*, 2013). The guard cell gene *SLAC1* has been identified as an important player in rapid stomatal responses to environmental factors (Negi *et al.*, 2008; Vahisalu *et al.*, 2008; Merilo *et al.*, 2013). Elevated bicarbonate, more so than elevated [CO_2_], activates the intracellular free calcium ion [Ca^2+^]_i_ sensitivity of SLAC1-mediated anion channels, thus reducing stomatal opening (Xue *et al.*, 2011). In this study, *NR* silencing significantly reduced elevated [CO_2_]-induced NO accumulation and stomatal closure, which was reversed by SNP application, whereas *SLAC1* silencing also blocked stomatal closure, but could not be reversed by exogenous SNP (Fig. 4). These results indicate that both NR and SLAC1 play crucial roles in elevated [CO_2_]-induced stomatal closure (Bright *et al.*, 2006; Negi *et al.*, 2008; Xue *et al.*, 2011), but NR acts in an NO-dependent manner, whereas SLAC1 acts in an NO-independent manner. The increased stomatal opening in *NR*- and *SLAC1*-silenced plants resulted in significant increases in *P. syringae* infection and disease expression, and also blocked elevated [CO_2_]-induced *P. syringae* defence to varying extents (Fig. 5), confirming the role for stomata in restricting bacterial entry into plant tissue. In this study, elevated [CO_2_] also inhibited COR-induced stomatal opening (Fig. 1D). How NO is involved in this process remains unclear. In a previous study on epidermal peels of *Arabidopsis* plants, even though NO is suggested to be required for PAMPs and bacteria to close stomata, COR did not block synthesis of NO induced by PAMPs perception; the researchers thus speculated that COR acts downstream or independently of NO synthesis to block stomatal closure (Melotto *et al.*, 2006). In a recent study on *Arabidopsis*, COR-induced decrease in photosynthetic efficiency at ambient CO_2_ was found to be eliminated by supplementation of plants with high CO_2_ for only a 2-h period, suggesting atmospheric [CO_2_] had fundamental effects on COR-mediated function (Attaran *et al.*, 2014). Thus, further studies will be necessary to determine the precise effect mode of elevated [CO_2_] on COR-mediated stomatal movement.

*SLAC1*-silenced plants, however, grown under elevated [CO_2_] still had significantly higher resistance compared with those grown under ambient [CO_2_] (Fig. 5), implying that factors other than the stomata also play a role in elevated [CO_2_]-induced *P. syringae* resistance. In accordance, we also subjected tomato plants to *P. syringae* inoculation by syringe infiltration method. In such condition, entry of *P. syringae* through stomata is not a restricting factor, and we found that elevated [CO_2_] plants still had higher defence level than that of ambient [CO_2_] counterpart (Fig. 6), verifying the results obtained on *NR*- and *SLAC1*-silencing plants. In addition, as the NO content was higher in the elevated [CO_2_]-treated *SLAC1*-silenced plants when compared with the ambient counterpart (Fig. 4), we speculate that NO-related physiological events other than stomatal closure also contribute to the elevated [CO_2_]-induced resistance. NO is now recognised as a crucial player in plant defence against pathogens. Many proteins that are specifically regulated by NO and that participate in signalling during the plant defence response have been identified, highlighting the physiological relevance of these modifications in plant immunity (Bellin *et al.*, 2013). Alternatively, other unknown factors may also be involved in elevated [CO_2_]-induced *P. syringae* resistance, thus underscoring the need for further investigation.

In conclusion, we found that the susceptibility of tomato plants to *P. syringae* is reduced under elevated [CO_2_] conditions. Here, we propose a dual mechanism involving elevated [CO_2_]-induced reductions in stomata-dependent and -independent pathways underlying this elevated [CO_2_]-induced defence response. This information is important for making proper predictions with regard to disease pressure and for designing strategies to improve plant pathogen resistance, especially to foliar bacterial pathogens, under changing agricultural and natural ecosystems.

## Abbreviations:

cfu: colony-forming units
[CO_2_]: CO_2_ concentration
COR: coronatine
hpi: hours post inoculation
NO: nitric oxide
NR: nitrate reductase
PAMPs/MAMPs: pathogen/microbe-associated molecular patterns
SEM: scanning electron microscopy
SLAC1: guard cell slow-type anion channel 1
TRV: tobacco rattle virus
VIGS: virus-induced gene silencing
ΦPSII: photochemical quantum yield at photosystem II.
